# Prediction of breast cancer-related lymphedema risk after postoperative radiotherapy *via* multivariable logistic regression analysis

**DOI:** 10.3389/fonc.2022.1026043

**Published:** 2022-10-26

**Authors:** Jae Sik Kim, Jin Ho Kim, Ji Hyun Chang, Do Wook Kim, Kyung Hwan Shin

**Affiliations:** ^1^ Department of Radiation Oncology, Seoul National University College of Medicine, Seoul, South Korea; ^2^ Department of Radiation Oncology, Soonchunhyang University Seoul Hospital, Seoul, South Korea; ^3^ Department of Radiation Oncology, Seoul National University Hospital, Seoul, South Korea

**Keywords:** breast cancer, lymphedema, prediction model, radiotherapy, regional nodal irradiation, taxane

## Abstract

**Purpose:**

We identified novel clinical and dosimetric prognostic factors affecting breast cancer-related lymphedema after postoperative radiotherapy (RT) and developed a multivariable logistic regression model to predict lymphedema in these patients.

**Methods and materials:**

In total, 580 patients with unilateral breast cancer were retrospectively reviewed. All patients underwent breast surgery and postoperative RT with or without systemic treatment in 2015. Among the 580 patients, 532 with available RT plan data were randomly divided into training (n=372) and test (n=160) cohorts at a 7:3 ratio to generate and validate the lymphedema prediction models, respectively. An area under the curve (AUC) value was estimated to compare models.

**Results:**

The median follow-up duration was 5.4 years. In total, 104 (17.9%) patients experienced lymphedema with a cumulative incidence as follows: 1 year, 10.5%; 3 years, 16.4%; and 5 years, 17.6%. Multivariate analysis showed that body mass index ≥25 kg/m^2^ (hazard ratio [HR] 1.845), dissected lymph nodes ≥7 (HR 1.789), and taxane-base chemotherapy (HR 4.200) were significantly associated with increased lymphedema risk. Conversely, receipt of RT at least 1 month after surgery reduced the risk of lymphedema (HR 0.638). A multivariable logistic regression model using the above factors, as well as the minimum dose of axillary level I and supraclavicular lymph node, was created with an AUC of 0.761 and 0.794 in the training and test cohorts, respectively.

**Conclusions:**

Our study demonstrated that a shorter interval from surgery to RT and other established clinical factors were associated with increased lymphedema risk. By combining these factors with two dosimetric parameters, we propose a multivariable logistic regression model for breast cancer-related lymphedema prediction after RT.

## Introduction

Ipsilateral upper extremity lymphedema is a chronic and progressive sequela that occurs after breast cancer treatment. The incidence of breast cancer-related lymphedema is reportedly 38.2%, in the case of patients with regional nodal irradiation (RNI) (1). This severe complication diminishes the patient’s quality of life both physically and psychologically (2). Patients with lymphedema experience arm swelling, stiffness, pain, and altered body image (2). Consequently, these patients show elevated psychological distress compared to patients without lymphedema (3).

For radiotherapy (RT), RNI has both positive and negative effects. RNI offers robust regional control, decreasing the subsequent risk of distant metastasis and breast cancer death (4). RNI is also a well-known risk factor for lymphedema: the risk of lymphedema is 1.3-fold higher in patients who receive RNI after axillary lymph node dissection (ALND) than in patients who do not receive RNI (5-year lymphedema incidence, 31.2% vs. 24.6%) (5). This increased risk might result from radiation and chronic inflammation-induced fibrosis (6).

Lymphedema progression can be prevented if early diagnosis and appropriate intervention are performed in the reversible phase (7). Accordingly, considerable research has been conducted to determine risk factors for lymphedema and to develop nomograms or scoring systems for the identification of high-risk patients (8–15). Radiation doses to specific regions of the regional nodal area increase the risk of lymphedema (9, 16, 17). However, while the mean lung dose and lung volume receiving ≥20 Gy are important for predicting RT pneumonitis after thoracic RT (18), there are no established RT dosimetric parameters for the determination of lymphedema risk. In several proposed prediction models, RT field design (breast/chest wall [CW] and/or RNI) or fractionation (conventional vs. hypofractionated) has been included as a variable (8–10, 12, 14, 15, 19); to our knowledge, no lymphedema prediction model uses detailed RT dosimetric parameters.

In this study, we estimated the cumulative incidence of lymphedema and the semi-annual conditional lymphedema-free rate in breast cancer patients receiving postoperative RT. We explored specific prognostic factors associated with RT and generated a novel prediction model for lymphedema development within 3 years after postoperative RT in these patients.

## Methods and materials

### Study population

This study was approved by the institutional review board at Seoul National University Hospital (No. H-2103-077-1204). The requirement for informed consent was waived because of the retrospective study design and limited risk to patients. The study was performed in accordance with the Declaration of Helsinki.

Women with ductal carcinoma *in situ* or invasive breast cancer who underwent breast surgery in 2015 at Seoul National University Hospital were included in the study. All patients received postoperative RT. We excluded patients with at least one of the following characteristics: initial M1 stage, previous history of RT to the CW, partial breast irradiation or partial completion of planned RT, other malignancies including contralateral breast cancer, locoregional failure and/or distant metastasis, and follow-up duration less than 1 year after RT. Finally, 580 patients were analyzed. Because we aimed to generate a prediction model using detailed RT dosimetric parameters, 532 patients with available RT plan data were used for model development. Random sampling with a 7:3 ratio was performed to divide these patients into training (n=372) and test (n=160) cohorts (**Table A1**).

### Treatment

Patient treatment strategies were determined according to their breast cancer stage and tumor molecular subtypes (**Table 1**). In most patients (n=490, 84.5%), breast-conserving surgery was performed. In terms of lymph node (LN) surgery, sentinel LN biopsy and ALND constituted 78.4% (n=455) and 20.9% (n=121) of cases, respectively, with a median of seven dissected LNs. Two hundred seventeen (37.4%) patients did not receive chemotherapy; neoadjuvant taxane-based chemotherapy was administered to 161 (27.8%) patients, while taxane-based regimens in the adjuvant setting were administered to 127 (21.9%) patients. Seventy-five (12.9%) patients were treated with non-taxane agents.

**Table 1:** Baseline characteristics of all patients (n = 580).

Variables	Total (n = 580)
Body mass index (kg/m^2^)	
< 25	409 (70.5)
25–30	147 (25.3)
> 30	24 (4.1)
Mammographic breast density	
1–2	102 (17.6)
3–4	478 (82.4)
T stage^*^	
Tis	16 (2.8)
1–2	522 (90.0)
3–4	42 (7.2)
N stage^*^	
0	364 (62.8)
1–3	216 (37.2)
Breast surgery	
Breast-conserving	490 (84.5)
Total mastectomy	90 (15.5)
LN surgery^†^	
Sentinel LN biopsy	455 (78.4)
Axillary LN dissection	121 (20.9)
Chemotherapy	
Neoadjuvant taxane	161 (27.8)
Adjuvant taxane	127 (21.9)
Non-taxane	75 (12.9)
Herceptin	101 (17.4)
Endocrine therapy	
Tamoxifen	280 (48.3)
Aromatase inhibitor	157 (27.1)
Radiotherapy fractionation^‡^	
Conventional	185 (31.9)
Fraction size (Gy, median)	1.8 (1.8–2.0)
Total dose (Gy, median)	50.4 (50.0–50.4)
Hypofractionated	395 (68.1)
Fraction size (Gy, median)	2.7 (2.4–3.0)
Total dose (Gy, median)	43.2 (39.0–48.6)
Regional nodal irradiation^‡^	178 (30.7)
Conventional	64 (36.0)
Fraction size (Gy, median)	1.8
Total dose (Gy, median)	50.4
Hypofractionated	114 (64.0)
Fraction size (Gy, median)	2.7 (2.4–2.7)
Total dose (Gy, median)	43.2 (43.2–48.6)

All variables are presented as n (% or range), unless otherwise stated.

^*^Clinical stage for patients with neoadjuvant chemotherapy and pathologic stage for others.

^†^Median number of dissected lymph nodes: 7 (range, 1–41).

^‡^Prescription dose. LN, lymph node.

Three-dimensional conformal RT was delivered with the following fractionation scheme by two radiation oncologists independently: one performed conventional (n=185, 31.9%) RT, and the other used hypofractionated (n=395, 68.1%). For conventional fractionation, breast/CW received a total median dose of 50.4 Gy (daily dose of 1.8 Gy). Hypofractionated RT with a total median dose of 43.2 Gy in 16 fractions was prescribed to the breast/CW. RNI was conducted in 178 (30.7%) patients with an identical scheme of breast/CW dose. All patients with conventional RNI received an equivalent dose in 2 Gy (EQD2, α/β=3) of 48.4 Gy. However, EQD2 ranging from 49.2 to 55.4 Gy was delivered to patients with hypofractionated RNI: 49.2 Gy (n=64), 51.8 Gy (n=1), 52.3 Gy (n=5), and 55.4 Gy (n=4). Additional 2-dimensional electron boost (9–14 Gy) to the tumor bed was delivered to 486 (83.8%) patients. Because the electron boost plan could not be technically applied to the three-dimensional conformal RT plan and its effect on the dose distribution of regional LNs was minimal, we did not consider the electron boost in dosimetric analysis. The median interval from surgery to RT was 1.1 (interquartile range, 0.9–1.2) and 6.4 (4.9–7.1) months in patients without or with adjuvant chemotherapy, respectively.

### Lymphedema

Lymphedema was assessed both by patient self-reporting and by physical examination at regular intervals during follow-up visits. Patients with suspected breast cancer-related lymphedema were referred to cancer rehabilitation specialists. Lymphedema was diagnosed when the difference in arm circumference between the affected and contralateral arm was >2 cm using a previously described assessment technique, or when a volume difference of >200 mL or >10% between them was detected using a perometer (20, 21). The cancer rehabilitation specialists diagnosed lymphedema after careful evaluation of the patient’s condition and started lymphedema management. Lymphedema occurring within 3 months after breast cancer surgery was excluded to distinguish transient arm swelling related to surgery or chemotherapy (5).

### Mammographic breast density

Breast density on initial mammography images was scored by breast radiologists based on the Breast Imaging-Reporting and Data System, ranging from 1 (lowest) to 4 (highest) (10, 22).

### RT dosimetric parameters

During RT planning, we did not routinely contour each axillary level ‘separately’; the axillary was contoured as a whole region along with breast/CW and we did not regard them as organs at risk. Retrospectively, the clinical target volume of axillary levels I, II, and III, as well as supraclavicular lymph nodes (SCLs)—all identified using the Radiation Therapy Oncology Group atlas—were automatically contoured (Figure A1) to the initial simulation computed tomography scans of 532 patients using the AVIEW RT ACS (Corelinesoft, Seoul, Korea) (23, 24). After calculation of the absorbed dose of auto-contoured regions, dose-volume histogram metrics of ipsilateral nodes were extracted and converted into the EQD2 with α/β=3. The following dosimetric parameters were obtained in each nodal region (distributions are shown in Figure A2): minimum dose (Dmin); maximum dose (Dmax); mean dose (Dmean); and relative volume (in percentage) receiving ≥10 Gy (V10), ≥20 Gy (V20), ≥30 Gy (V30), ≥40 Gy (V40), ≥50 Gy (V50), ≥60 Gy (V60), and ≥70 Gy (V70).

### Statistical analyses

The cumulative incidence of lymphedema from RT completion to lymphedema diagnosis was depicted using the Kaplan–Meier method. The cumulative incidences of lymphedema according to the RT plan were compared using the log-rank test. The 6-month conditional lymphedema-free rate was defined as the probability of remaining lymphedema-free for 6 months, given that a patient did not have lymphedema at the beginning of that period (25). Cox proportional hazards models were used to identify prognostic factors for lymphedema development in univariate and multivariate analyses. Variables with p<0.100 in univariate analysis were used for multivariate analyses after the multicollinearity check.

We used the training cohort (n=372) to generate several multivariable logistic regression models for prediction of lymphedema diagnosis until 3 years after postoperative RT. In the clinical model, four variables were included; all were significant prognostic factors for lymphedema in the above multivariate analysis. All dosimetric parameters had a p-value of <0.05 in the univariate logistic regression analysis (data are not shown); we selected dosimetric parameters with variance inflation factors under 5 to eliminate multicollinearity. Then, we used backward elimination to choose dosimetric parameters for the final multivariable logistic model. Clinical + dosimetric model I used variables in either the clinical or dosimetric model. Furthermore, we used random forest classification for variable importance measurements; we selected the best two predictors from clinical + dosimetric model I to establish clinical + dosimetric model II, a more simplified model. Each model performance was measured by the area under the curve (AUC) and the Akaike information criterion (AIC); it was then validated in the test cohort (n=160).

All statistical analyses were performed with R statistical software version 4.1.2 (https://www.r-project.org/). A two-sided p-value <0.05 was considered statistically significant.

## Results

### Baseline characteristics and lymphedema

**Figure 1:**
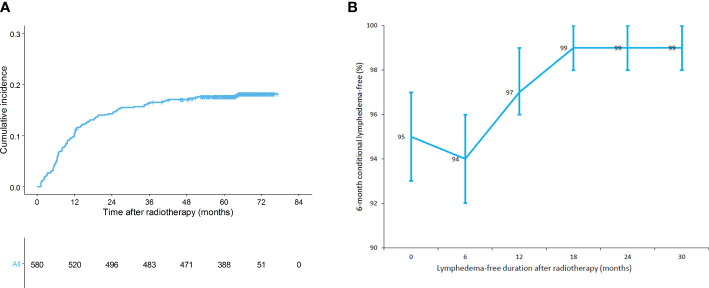
**(A)** Cumulative incidence of lymphedema (n = 580) and **(B)** 6-month conditional lymphedema-free rate (n = 580).

The median follow-up duration was 5.4 years (interquartile range, 5.1–5.7) and all patients remained disease-free: in our study period, only 85 patients were excluded because they had experienced treatment failures. The baseline characteristics of all patients are shown in **Table 1**. Most patients had low body mass index (BMI; <25 kg/m^2^) and high breast density. Ninety-eight patients underwent both ALND and RNI. Overall, 104 (17.4%) patients were diagnosed with lymphedema during follow-up (**Figure 1A**). The respective 1-, 3-, and 5-year cumulative incidences of lymphedema were 10.5%, 16.4%, and 17.6%, indicating that the lymphedema development plateau had been reached. The 6-month conditional lymphedema-free rate is shown in **Figure 1B**. If lymphedema did not occur within 12 months after RT, there was a 97% likelihood (95% confidence interval [CI]: 96–99%) that lymphedema would not develop for the next 6 months. There was no difference in lymphedema incidence between patients who received conventional RT and patients who received hypofractionated RT (14.0% vs. 17.5% at 3 years, p=0.300).

### Ascertainment of prognostic factors for developing lymphedema

**Table 2** shows the univariate and multivariate analyses of factors that affected lymphedema development throughout the study population. Multivariate analysis showed that higher BMI (≥25 kg/m^2^, hazard ratio [HR]: 1.845, 95% CI: 1.249–2.726; p=0.002), a large number of dissected LNs (≥7, HR: 1.789, 95% CI: 1.137–2.814; p=0.012), and receipt of taxane-based chemotherapy (compared with no chemotherapy; HR: 4.200, 95% CI: 1.982–8.901; p<0.001) significantly increased the risk of lymphedema. A longer interval from surgery to RT (≥1 month) decreased lymphedema development with an HR of 0.638 (95% CI: 0.411–0.990; p=0.045). In the RNI group, higher EQD2 (>49 Gy) was marginally associated with an increased risk of lymphedema (HR: 1.639, 95% CI: 0.942–2.850; p=0.080). In our analysis, mammographic breast density was not associated with lymphedema development (p=0.138).

**Table 2:** Univariate and multivariate analyses of factors associated with lymphedema development (n = 580).

	Univariate analysis		Multivariate analysis^*^	
Variables	HR (95% CI)	p-value	HR (95% CI)	p-value
Age ≥ 50 years	0.783 (0.530–1.155)	0.217		
Body mass index ≥ 25 kg/m^2^	1.982 (1.344–2.921)	< 0.001	1.845 (1.249–2.726)	0.002
Mammographic breast density (incremental)	0.822 (0.635–1.065)	0.138		
T stage 3-4	2.731 (1.602–4.655)	< 0.001	1.212 (0.666–2.206)	0.530
Total mastectomy	2.736 (1.804–4.151)	< 0.001	1.048 (0.635–1.729)	0.854
Number of dissected LNs ≥ 7	2.516 (1.623–3.900)	< 0.001	1.789 (1.137–2.814)	0.012
Chemotherapy				
Taxane-based (vs. no)	7.524 (3.909–14.482)	< 0.001	4.200 (1.982–8.901)	< 0.001
Non-taxane (vs. no)	2.045 (0.778–5.372)	0.147	2.018 (0.737–5.524)	0.172
Endocrine therapy				
Tamoxifen (vs. no)	1.044 (0.663–1.643)	0.853		
Aromatase inhibitor (vs. no)	0.626 (0.353–1.111)	0.110		
Herceptin administered	1.541 (0.977–2.432)	0.063	0.904 (0.562–1.454)	0.677
Interval between surgery and radiotherapy ≥ 1 month	0.702 (0.461–1.068)	0.099	0.638 (0.411–0.990)	0.045
Hypofractionated radiotherapy	1.253 (0.816–1.924)	0.302		
Regional nodal irradiation^†^				
No (vs. regional nodal irradiation EQD2 ≤ 49 Gy)	0.283 (0.160-0.499)	<0.001	0.633 (0.326-1.229)	0.177
Regional nodal irradiation EQD2 > 49 Gy (vs. ≤ 49 Gy)	1.758 (1.026–3.009)	0.040	1.639 (0.942–2.850)	0.080

^*^Variables with p<0.100 in univariate analysis were used.

^†^Prescription dose. CI, confidence interval; EQD2, equivalent dose in 2 Gy (α/β=3); HR, hazard ratio; LN, lymph node.

### Multivariable logistic regression models for lymphedema risk

**Figure 2:**
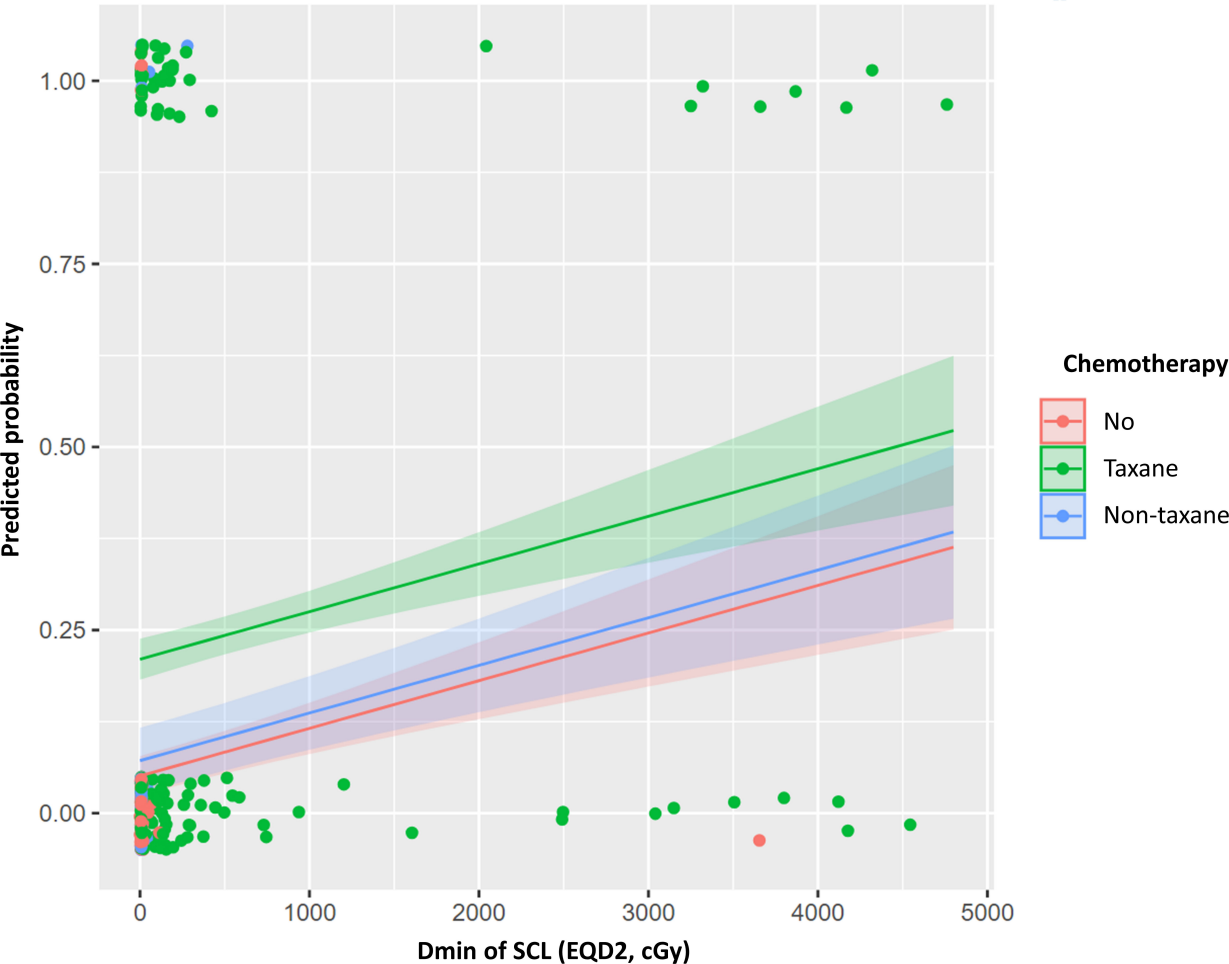
Predicted probability curve of clinical + dosimetric model II for lymphedema risk within 3 years after postoperative radiotherapy. Dmin, minimum dose; EQD2, equivalent dose in 2 Gy (α/β=3); SCL, supraclavicular lymph node.

We generated four models for prediction of lymphedema risk using only clinical or dosimetric factors and combinations of these factors (**Table 3**). The clinical model included BMI, number of dissected LNs, chemotherapy regimen, and the interval between surgery and RT. All variables were scored as 0 or 1 (Eq. A1). The AUCs of this model were 0.743 and 0.805 in the training and test cohorts, respectively. The selected dosimetric parameter-only model used a continuous Dmin of axillary level I and SCL; its AUCs were 0.692 and 0.710 in the training and test cohorts, respectively. We integrated the above two models to develop clinical + dosimetric model I. The model AUC was 0.761 in the training cohort; it exhibited enhanced fitness, as quantified by AIC. The model AUC was 0.794 in the test cohort. Among covariates in clinical + dosimetric model I, the chemotherapy regimen and Dmin of SCL were identified as the most predictive factors according to random forest classification. Using these factors, we developed clinical + dosimetric model II; the predicted probability curve of this model is shown in **Figure 2**. All equations of these models are presented in Equation A1.

**Table 3:** Comparison of multivariable logistic regression models for prediction of lymphedema risk within 3 years after postoperative radiotherapy.

		AUC		
Model	Predictors	Training	Test	AIC
Clinical	Body mass index (<25 vs. ≥25 kg/m^2^) No. of dissected lymph nodes (< 7 vs. ≥ 7) Chemotherapy regimen (no, taxane, non-taxane) Interval between surgery and radiotherapy (< 1 vs. ≥ 1 month)	0.743	0.805	247
Dosimetric	Dmin of axillary level I (EQD2, incremental) Dmin of supraclavicular lymph node (EQD2, incremental)	0.692	0.710	245
Clinical + Dosimetric I	Body mass index (<25 vs. ≥25 kg/m^2^) No. of dissected lymph nodes (< 7 vs. ≥ 7) Chemotherapy regimen (no, taxane, non-taxane) Interval between surgery and radiotherapy (< 1 vs. ≥ 1 month) Dmin of axillary level I (EQD2, incremental) Dmin of supraclavicular lymph node (EQD2, incremental)	0.761	0.794	238
Clinical + Dosimetric II^*^	Chemotherapy regimen (no, taxane, non-taxane) Dmin of supraclavicular lymph node (EQD2, incremental)	0.733	0.791	243

^*^This model uses chemotherapy regimen and Dmin of supraclavicular lymph node, which are the most two important predictors in clinical + dosimetric model I according to random forest classification. AIC, Akaike information criterion; AUC, area under the curve; Dmin, minimum dose; EQD2, equivalent dose in 2 Gy (α/β=3).

## Discussion

This study showed that approximately one in five breast cancer survivors developed lymphedema and had a high risk of lymphedema until 1 year after RT. Immediate RT following surgery was identified as a novel risk factor. To our knowledge, this is the first study to generate a model for prediction of lymphedema risk by using the dosimetric parameters of regional LNs and other clinical data. The clinical + dosimetric model I was the best in terms of AUC and AIC. This model could assist physicians in counseling their patients with high model fitness. Moreover, based on this model, we propose a simplified predicted probability curve of lymphedema risk (clinical + dosimetric model II); it could be used during RT planning, like the mean lung dose and V20 to predict radiation pneumonitis.

Our analysis revealed that a shorter period from surgery to RT was significantly associated with a higher incidence of lymphedema. Furthermore, lymphedema occurred rapidly within 1 year after RT in our cohort; the semi-annual conditional lymphedema-free rate also tended to be lower within 1 year. Additionally, this pattern was observed in patients with RNI, which is associated with late-onset lymphedema because of RT-induced fibrosis (5). This phenomenon might be explained by the damaging effects of RT on wound healing in the lymphatic system after surgery, rather than chronic RT-induced fibrosis. Indeed, a meta-analysis showed that the Lymphatic Microsurgical Preventive Healing Approach, conducted alongside ALND, significantly reduced lymphedema incidence by 23.1% in patients with ALND and RNI (26). Therefore, intensive management of damaged lymphatics might be required; an appropriate start time should be considered if RT is planned.

We confirmed that BMI, the extent of LN harvest, and taxane-based chemotherapy were associated with lymphedema development, consistent with findings in previous studies (5, 9, 10, 12–16, 19). High BMI indicates both that patients have more subcutaneous tissue in limbs, serving as a reservoir for interstitial fluid, and that they need more extensive surgery due to subcutaneous fat (27). Lymph node dissection and BMI, to some extent, are associated with lymphatic destruction. Taxane, especially docetaxel, enhances conditions conducive to lymphedema by interstitial fluid filtration and capillary protein leakage (28). It was recently revealed that low breast density in mammography increases the risk of lymphedema development (10); breast density is a surrogate for impaired fat metabolism and poor lymphatic vasculature function (10). However, we failed to demonstrate a relationship between mammographic breast density and lymphedema. This discrepancy should be investigated in future studies. Because mammography is routinely used for breast cancer screening and diagnosis, future prospective breast cancer studies could readily include mammographic breast density and lymphedema toxicity.

In univariate analysis, high EQD2 of the regional node was associated with increased lymphedema; this association showed marginal significance after adjustments for other prognostic factors. This finding might reflect the EQD2 distribution. Most patients assigned to the EQD2 >49 Gy group were treated with 49.2 Gy, which differed by 0.8 Gy from the dose in patients treated with ≤49 Gy. Although RNI EQD2 was not an independent factor in our analysis, the receipt of RT is a well-known risk factor for lymphedema; thus, RT dosimetric parameters were investigated in detail. It has been reported that Dmin of the axillary-lateral thoracic vessel juncture is a significant predictor, suggesting that specific regions of irradiation might be more important than the overall dose (17). Consistent with that notion, multivariate analysis with dosimetric parameters alone found that Dmin of axillary level I and SCL had a significant effect on lymphedema development.

Although the tumor was well-controlled within a median of five years in this study, we should caution against interpreting that lymphedema should be reduced by lowering the dose of these regions. Instead, we recommend that physicians try to make the best RT delivery plan with acceptably low doses in these areas, without compromising dose coverages. If it is not feasible, physicians should identify high-risk patients with lymphedema based on our model and provide them with proper management.

AIC values indicated that clinical + dosimetric model I was superior to models established with either clinical or dosimetric data alone; this highlights two important considerations. First, the strong lymphedema-associated dosimetric parameter was Dmin. Considering that the capillary bed and lymphatics involved in the production and circulation of lymph fluid are spread over the contoured nodal area (29), a dose covering 100% of volume might have substantial effects. Second, Dmin of SCL, instead of axillary level I, was identified as a more robust predictive factor. This finding was consistent with several other studies (30–32). Some studies have demonstrated that the exclusion of upper axillary levels I and II decreases lymphedema probability (9, 16). Furthermore, an anatomical study identified the axillary-lateral thoracic vessel juncture, superior to axillary level I, as an organ at risk of lymphedema in patients with breast cancer (17). These reports indicate that the upper axillary regions are significantly and independently associated with lymphedema occurrence; in this context, Dmin of SCL might indirectly indicate the dose of these areas.

Our results had several potential limitations. First, this study had a retrospective design and was conducted at a single institution with a small sample size: the population was very varied in terms of the treatments received, despite the pragmatic approach. Multi-center studies are needed for external validation of our prediction model; our findings should also be assessed in prospective trials. Second, arm circumference measurement was used to detect lymphedema. This method is widely used but might involve interrater variability. In addition, the lack of bilateral arm circumference values before breast cancer surgery might create difficulty differentiating between pre-existing arm asymmetries and lymphedema; this could bias the observed incidence of lymphedema. However, considering a preoperative baseline measurement was not routinely performed, our results could reflect real-world practice. Lastly, the effect of RT fractionation, which is presumed to affect the risk of lymphedema, could not be elucidated in this study.

With the increasing number of breast cancer survivors (33), lymphedema has emerged as an increasingly important issue; proper management (including preventive strategies) is necessary. This study demonstrated that lymphedema development is more likely during the first year after RT; there is a need for cautious monitoring at appropriate intervals. Radiation oncologists should determine the start date of RT after careful consideration of previous breast cancer surgery and the recovery of lymphatic function, especially in patients at high risk of lymphedema. To identify patients at high risk of lymphedema, we suggest a model for predicting the 3-year lymphedema rate using patient-related and treatment-related factors.

## Data availability statement

The datasets generated and/or analyzed during the current study are available from the corresponding author on reasonable request.

## Ethics statement

The studies involving human participants were reviewed and approved by the Institutional Review Board at Seoul National University Hospital (No. H-2103-077-1204). Written informed consent for participation was not required for this study in accordance with the national legislation and the institutional requirements.

## Author contributions

Conceptualization–JSK and KHS; Supervision–KHS; Data curation–JSK, JHK, JHC, and DWK; Methodology–JSK and KHS; Formal analysis and visualization–JSK and KHS; Writing-original draft–JSK; Writing-review and editing–JSK, JHK, JHC, DWK, and KHS. All authors have read and agreed to the published version of the manuscript.

## Acknowledgments

We would like to express our appreciation to Corelinesoft, a medical artificial intelligence company, for their assistance with auto-contouring with AVIEW RT ACS.

## Conflict of interest

The authors declare that the research was conducted in the absence of any commercial or financial relationships that could be construed as a potential conflict of interest.

## Publisher’s note

All claims expressed in this article are solely those of the authors and do not necessarily represent those of their affiliated organizations, or those of the publisher, the editors and the reviewers. Any product that may be evaluated in this article, or claim that may be made by its manufacturer, is not guaranteed or endorsed by the publisher.
