# Supplementary material for: Prediction of breast cancer-related lymphedema risk after postoperative radiotherapy via multivariable logistic regression analysis
Source: Front Oncol. 2022 Oct 26;12:1026043. doi: 10.3389/fonc.2022.1026043 (PMC9643832; doi:10.3389/fonc.2022.1026043)
Supplement: Supplementary file 1 [file DataSheet_1.docx]

Supplementary Material

**Table A1.** Baseline characteristics of training and test cohort (n=532).

| **Variables** | **Training**  **(n=372)** | **Test**  **(n=160)** | **p-value^*^** |
| --- | --- | --- | --- |
| Age (year, median) | 48.5 (25–80) | 49 (26–76) | 0.373 |
| Body mass index (kg/m^2^) |  |  | 0.847 |
| < 25 | 264 (71.0) | 112 (70.0) |  |
| 25–30 | 91 (24.5) | 42 (26.2) |  |
| > 30 | 17 (4.6) | 6 (3.8) |  |
| T stage^†^ |  |  | 0.969 |
| Tis | 10 (2.7) | 6 (3.8) |  |
| 1 | 196 (52.7) | 82 (51.2) |  |
| 2 | 146 (39.2) | 64 (40.0) |  |
| 3 | 18 (4.8) | 7 (4.4) |  |
| 4 | 2 (0.5) | 1 (0.6) |  |
| N stage^†^ |  |  | 0.564 |
| 0 | 255 (68.5) | 103 (64.4) |  |
| 1 | 69 (18.5) | 29 (18.1) |  |
| 2 | 40 (10.8) | 24 (15.0) |  |
| 3 | 8 (2.2) | 4 (2.5) |  |
| Breast surgery |  |  | 0.180 |
| Breast-conserving | 343 (92.2) | 141 (88.1) |  |
| Total mastectomy | 29 (7.8) | 19 (11.9) |  |
| LN surgery |  |  | 0.094 |
| Not done | 3 (0.8) | 1 (0.6) |  |
| Sentinel LN biopsy | 316 (84.9) | 124 (77.5) |  |
| Axillary LN dissection | 53 (14.2) | 35 (21.9) |  |
| Number of dissected LNs (median) | 7 (1-41) | 7 (1-26) | 0.304 |
| Neoadjuvant chemotherapy | 96 (25.8) | 44 (27.5) | 0.765 |
| Adjuvant chemotherapy | 126 (33.9)^‡^ | 52 (32.5) | 0.836 |
| Chemotherapy regimen |  |  | 0.571 |
| Not done | 151 (40.6) | 64 (40.0) |  |
| Neoadjuvant taxane | 94 (25.3) | 44 (27.5) |  |
| Adjuvant taxane | 72 (19.4) | 35 (21.9) |  |
| Non-taxane | 55 (14.8) | 17 (10.6) |  |
| Herceptin | 66 (17.7) | 22 (13.8) | 0.313 |
| Endocrine therapy |  |  | 0.816 |
| Not done | 98 (26.3) | 38 (23.8) |  |
| Tamoxifen | 176 (47.3) | 79 (49.4) |  |
| Aromatase inhibitor | 98 (26.3) | 43 (26.9) |  |
| Radiotherapy fractionation^§^ |  |  | 1.000 |
| Conventional | 129 (34.7) | 56 (35.0) |  |
| Fraction size (Gy, median) | 1.8 | 1.8 |  |
| Total dose (Gy, median) | 50.4 | 50.4 |  |
| Hypofractionated | 243 (65.3) | 104 (65.0) |  |
| Fraction size (Gy, median) | 2.7 | 2.7 |  |
| Total dose (Gy, median) | 43.2 | 43.2 |  |
| Regional nodal irradiation^§^ | 84 (22.6) | 50 (31.2) | 0.045 |
| Conventional | 40 (47.6) | 25 (50.0) |  |
| Fraction size (Gy, median) | 1.8 | 1.8 |  |
| Total dose (Gy, median) | 50.4 | 50.4 |  |
| Hypofractionated | 44 (52.4) | 25 (50.0) |  |
| Fraction size (Gy, median) | 2.7 | 2.7 |  |
| Total dose (Gy, median) | 43.2 | 43.2 |  |

All variables are presented as n (% or range), unless otherwise stated.

^*^p-value determined by chi-squared test or Fisher’s exact test for categorical variables and independent Student’s t-test for continuous variables.

^†^Clinical stage for patients with neoadjuvant chemotherapy and pathologic stage for others.

^‡^One patient received both neoadjuvant and adjuvant chemotherapies.

^§^Prescription dose.

Abbreviation: LN, lymph node.

**Figure A1.** Representative example of auto contouring of regional nodal areas based on the Radiation Therapy Oncology Group atlas.


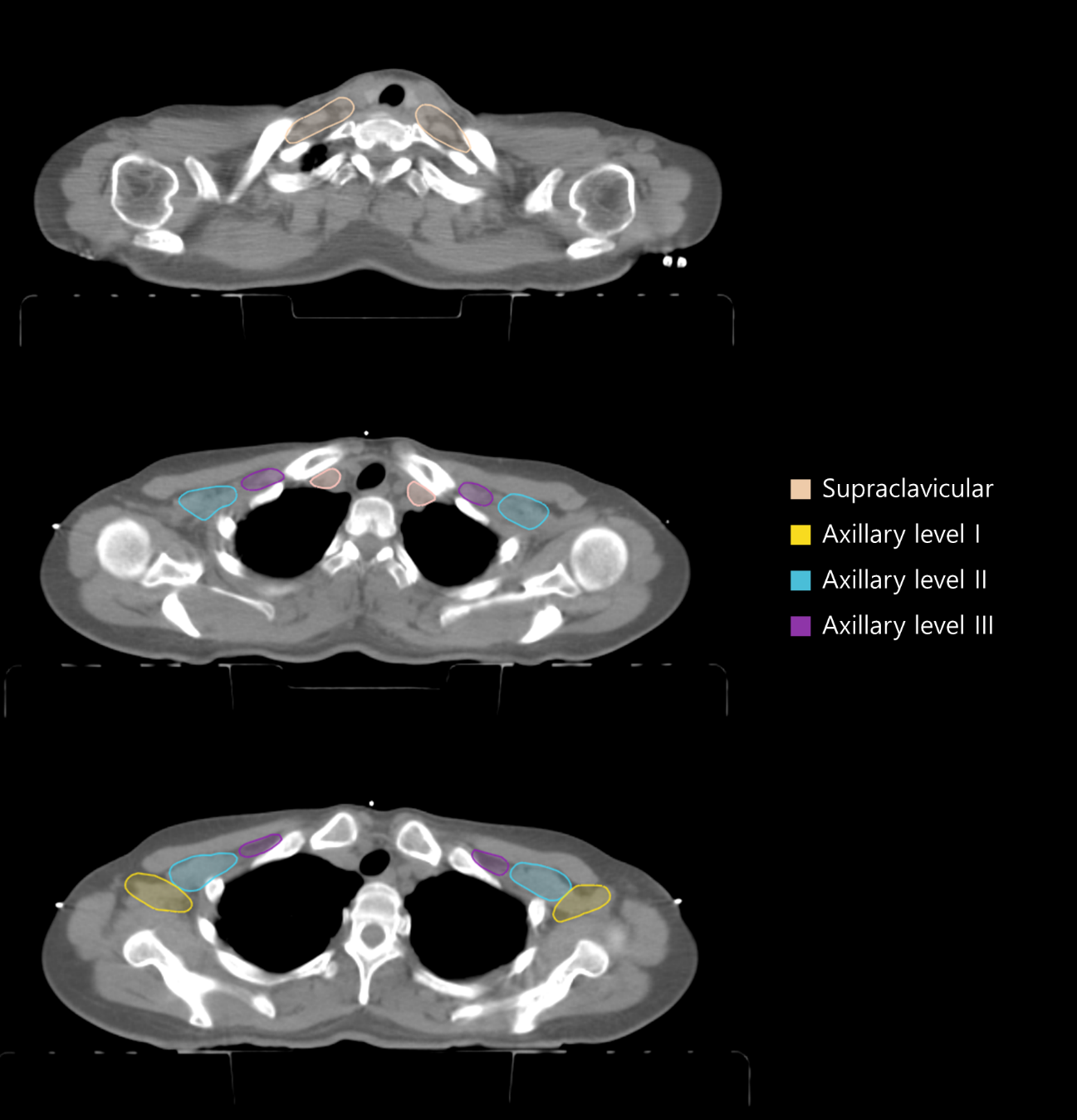


**Figure A2.** Summary of dose-volume histogram in each regional nodal area.

(A) Supraclavicular


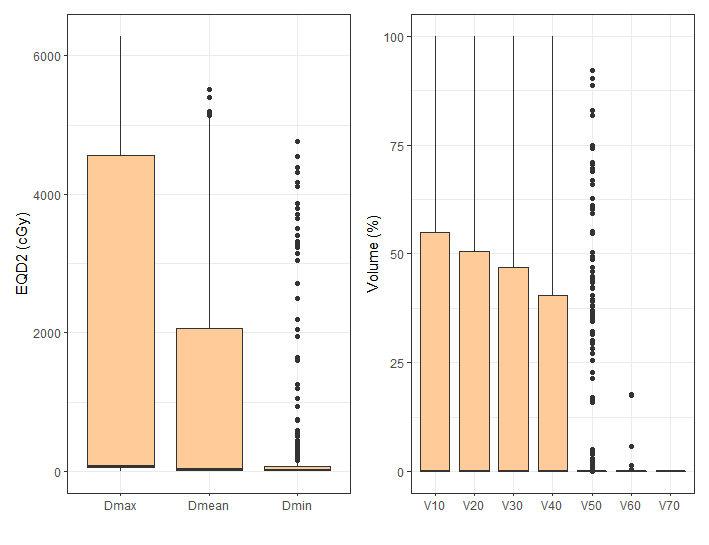


(B) Axillary level I


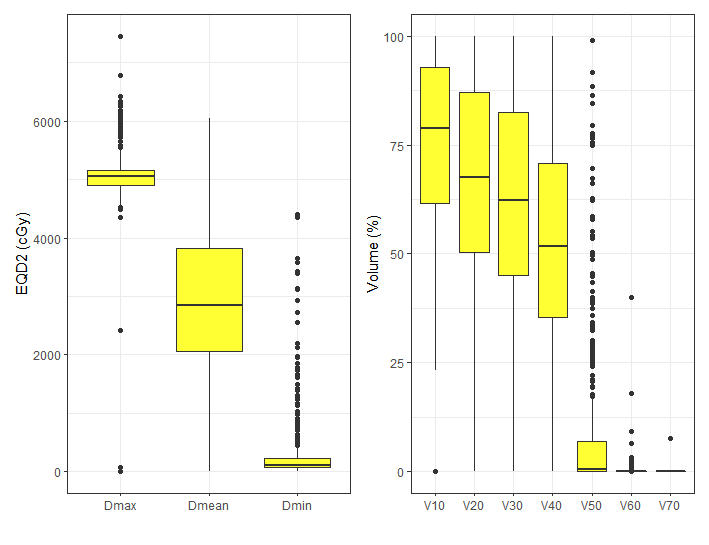


(C) Axillary level II


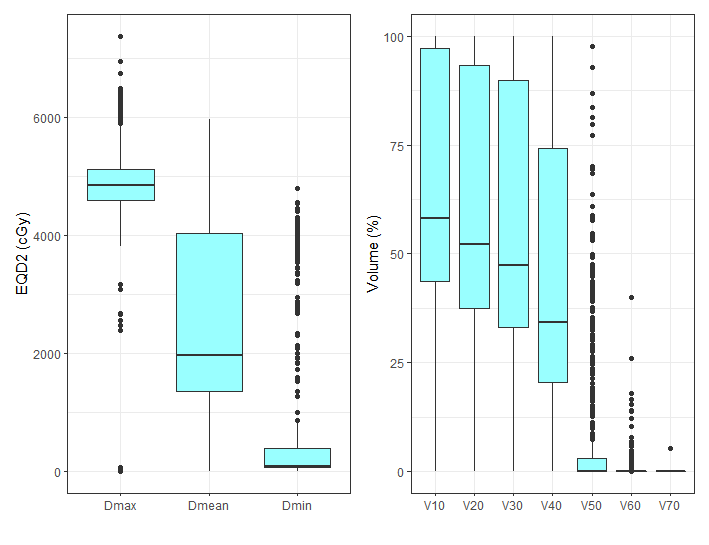


(D) Axillary level III


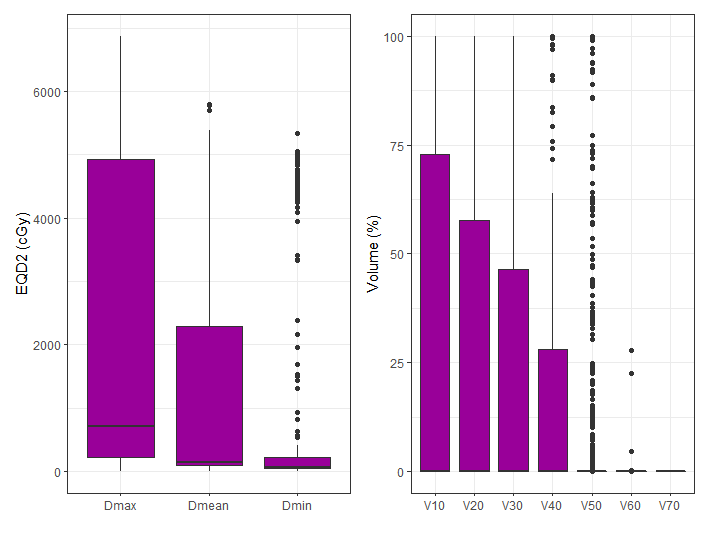


**Equation A1.** Mathematical formulations of multivariable logistic regression models for prediction of lymphedema risk within 3 years after postoperative radiotherapy. (a) Clinical, (b) dosimetric, (c) clinical + dosimetric I, and (d) clinical + dosimetric II models.

Define the following:

- P: probability of lymphedema
- BMI: body mass index (BMI) – 1 if BMI ≥ 25 kg/m^2^, 0 otherwise.
- LN: number of dissected lymph nodes – 1 if ≥ 7, 0 otherwise.
- CTx1: taxane chemotherapy – 1 if yes, 0 otherwise.
- CTx2: non-taxane chemotherapy – 1 if yes, 0 otherwise.
- I: interval between surgery and radiotherapy – 1 if ≥ 1 month, 0 otherwise.
- Ax1: minimum dose of axillary level I (EQD2, Gy).
- SCL: minimum dose of supraclavicular lymph node (EQD2, Gy).
  - EQD2: equivalent dose in 2 Gy (α/β = 3).

(A) Clinical model for lymphedema

$\log\left( \frac{P}{1-p} \right) = 0.063+(0.058*BMI)+(0.057*LN)+(0.188*CTx1)+(0.041*CTx2) -(0.079*I)$

(B) Dosimetric model for lymphedema

$\log\left( \frac{P}{1-p} \right) = 0.089+\left( 0.012*Ax1 \right)+\left( 0.008*SCL \right)$

(C) Clinical + dosimetric model I

$\log\left( \frac{P}{1-p} \right)=0.049+\left( 0.057*BMI \right)+\left( 0.047*LN \right)+\left( 0.134*CTx1 \right)+\left( 0.033*CTx2 \right)-\left( 0.065*I \right)+\left( 0.008*Ax1 \right)+ (0.005*SCL)$

(D) Clinical + dosimetric model II

$\log\left( \frac{P}{1-p} \right) = 0.051+(0.159*CTx1)+(0.021*CTx2)+(0.007*SCL)$
